# Canadian Diplomacy and Our Cattle-exports

**Published:** 1896-06

**Authors:** 


### CANADIAN DIPLOMACY AND OUR CATTLE-EXPORTS.

In these days of so-called diplomacy, one's friendship and respect for their colleagues of sister nations are greatly strained. Like charity, diplomacy covers a multitude of sins, and one is made at times to feel that he would like to see in operation the more direct means of accomplishing the ends to be served, because they are more honest and open.

Why our colleagues and friends in Canada will continue to try to prevent the exportation of American cattle from Canadian ports on the flimsy grounds that it endangers their market in certain countries, because of the imaginary existence of certain contagious and infectious diseases among our cattle, is too absurd, and flavors too strongly of dishonest and extremely selfish purposes.

If our growers of cattle are willing to accept smaller profits for their stock to the impairment of Canadian gains, why not be honest with us and so state it, and not attempt, under the cover of diplomacy, to cast a reflection upon our cattle-interests on the grounds of dangerous infectious and contagious diseases, that our professional *confréres* in Canada know to be untrue.

---

We would call the attention of our readers and the profession in general to several important and interesting local meetings of veterinary associations, all of which promise instructive gatherings as well as social pleasures and privileges, which in these trying times are the greatest safeguards in maintaining the true spirit and common purpose of our profession and renders more potent our force and influence in commanding more generous recognition and support at the hands of the people.

